# The epidemiology of cancer in the United Arab Emirates: A systematic review: Erratum

**DOI:** 10.1097/MD.0000000000014220

**Published:** 2019-01-11

In the article, “The epidemiology of cancer in the United Arab Emirates: A systematic review”,^[1]^ which appeared in Volume 97, Issue 50 if *Medicine*, Rami A. Ballout's degree appeared incorrectly and should be BS. Ballout's affiliation should be Faculty of Medicine, American University of Beirut, Beirut, Lebanon. Ballout's ORCID account should also be linked through https://orcid.org/0000-0002-7543-0895.
